# Feasibility and signal quality of the Minder® implantable continuous EEG monitoring® system compared to 10–20 scalp electrodes over extended monitoring periods

**DOI:** 10.1002/epi.70290

**Published:** 2026-05-20

**Authors:** Taneeta Mindy Ganguly, Alan Lai, Andrew Niemiec, Tracy Cameron, Erin C. Conrad, Ramya Raghupathi, Colin Ellis, Amy J. Halliday, Lisa Gillinder, Udaya Seneviratne, Patrick Kwan, Piero Perucca, Aileen McGonigal, Sara Vogrin, Holly Fontenot, Mark J. Cook

**Affiliations:** ^1^ Penn Neuroscience Center—Neurology Perelman Center for Advanced Medicine, University of Pennsylvania Philadelphia Pennsylvania USA; ^2^ Department of Medicine St. Vincents Hospital Melbourne, University of Melbourne Melbourne Victoria Australia; ^3^ Epiminder Pty Ltd Melbourne Victoria Australia; ^4^ Department of Clinical Neurosciences St Vincent's Hospital Melbourne Melbourne Victoria Australia; ^5^ Queensland Brain Institute The University of Queensland St Lucia Queensland Australia; ^6^ Neurology Department Princess Alexandra Hospital Brisbane Queensland Australia; ^7^ Department of Neurology Monash Medical Centre Melbourne Victoria Australia; ^8^ Neurology Department Alfred Health Melbourne Victoria Australia; ^9^ Department of Neurosciences, Central Clinical School Alfred Hospital, Monash University Melbourne Victoria Australia; ^10^ School of Public Health and Preventive Medicine Monash University Melbourne Victoria Australia; ^11^ Department of Medicine Austin Health, University of Melbourne Melbourne Victoria Australia; ^12^ Faculty of Medicine, Mater Hospital and Mater Research Institute, Mater Centre for Neurosciences The University of Queensland South Brisbane Queensland Australia

**Keywords:** detection, EEG, implant, seizure, subscalp

## Abstract

**Objective:**

To evaluate the signal quality of the Minder implantable continuous EEG monitoring (iCEM) system compared to traditional scalp EEG in the context of long‐term, continuous monitoring in individuals with epilepsy.

**Methods:**

Twenty‐six patients implanted with the Minder iCEM system (November 2019 to July 2023) underwent 7‐day video‐EEG co‐monitoring with full montage scalp EEG at 4 and 24 weeks post‐implantation. Co‐monitored EEG segments were rated for clarity on a 5‐point ordinal scale by blinded expert reviewers for ictal and interictal periods. Minder vs scalp ratings were compared using Wilcoxon signed‐rank test; 4‐ vs 24‐week comparisons used Wilcoxon rank‐sum test. Objective quantitative EEG analysis of the signal bandwidth was performed by application of spectral coherence analysis and Pearson's correlation coefficients.

**Results:**

No significant difference was found between interictal signal clarity reviewer ratings for Minder vs scalp EEG (*p* = .97), and there was no statistically significant difference between the Minder signal clarity ratings at 4 vs 24 weeks (*p* = .95). Pearson's correlation between Minder vs scalp EEG also indicated a high degree of similarity (median correlation = .69) and no statistically significant difference between correlations at 4 vs 24 weeks (*p* = .99). All electrographic seizures identified on full 10–20 scalp EEG were also identified on Minder; however, the full 10–20 montage received statistically higher seizure clarity ratings than Minder (*p* < .001).

**Significance:**

This study supports the feasibility of sub‐scalp EEG as a reliable long‐term monitoring tool. Interictal signal quality was statistically equivalent to limited‐montage scalp EEG, and all seizures identified on full 10–20 scalp EEG were also visualized on Minder. The full 10–20 montage did receive statistically higher seizure clarity ratings, reflecting its greater spatial resolution. The signal quality was stable through 24 weeks, demonstrating the fidelity of the device. These findings position the Minder system as a complement to full montage scalp EEG, particularly in ambulatory settings where continuous long‐term recording is required.

**Key points**

- Twenty‐six patients with epilepsy underwent Minder implantation with video/scalp EEG co‐monitoring at 4 and 24 weeks post‐implantation.
- Interictal signal quality was equivalent to limited‐montage scalp EEG by blinded board‐certified neurologist ratings.
- All seizures on full 10–20 scalp EEG were visualized on Minder; 10–20 did receive statistically higher seizure clarity ratings.
- High spectral coherence and signal correlation at 4 and 24 weeks confirmed long‐term functional integrity of the Minder system.
- This study supports the Minder iCEM system as a complement to 10–20 EEG for continuous bilateral monitoring with long‐term device fidelity.

## 1 INTRODUCTION

Nearly 52 million people have epilepsy worldwide.^1^ Epilepsy is characterized by the potential for recurrent seizures, and the seizures themselves are associated with substantial morbidity and mortality.^2^, ^3^, ^4^, ^5^, ^6^ Seizures are largely unpredictable in nature and have variable presentations, and many patients with seizures do not have access to the expertise required to accurately diagnose epilepsy and its mimickers.^7^ Despite the widespread nature and seriousness of the condition, epilepsy poses a significant diagnostic challenge, which currently lacks an easily accessible solution.

Accurate seizure count is essential to optimal treatment of patients. Self‐reporting of seizures by patients and caregivers remains the only broadly available method of obtaining seizure count. These self‐reported seizure diaries are widely regarded to be unreliable—less than 50% of seizures are reported by patients, and there is significant variability in seizure reporting over time.^8^ Conclusions drawn from seizure diaries may not accurately reflect a patient's disease state and pose considerable risk, as individual seizures may be life‐threatening.^9^

Our current standard of practice has limited characterization of ongoing seizures, which also poses noteworthy impacts on healthcare resources. More than 30% of patients with epilepsy continue to have seizures despite best medical management. Direct health care spending for the 3.4 million individuals with epilepsy and seizures was estimated to be approximately $24.5 billion annually.^10^, ^11^ Due to the recurrent nature of epilepsy and epilepsy mimickers such as psychogenic nonepileptic events, epilepsy is characterized by frequent utilization of high‐acuity medical settings—functional neurological disorders comprise up to 25% of patients referred to epilepsy specialist centers and incur an annual direct cost of $1.2 billion, and these costs have been demonstrated to reduce considerably after the diagnosis of functional neurological disorders (FND).^12^, ^13^ High‐fidelity extended long‐term ambulatory EEG has the potential to differentiate epileptic seizures from psychogenic nonepileptic events, assess seizure burden to gauge response to intervention and streamline therapies, and classify seizures for drug‐refractory patients.

There is a growing need for long‐term, continuous EEG‐monitoring systems that can provide a more comprehensive picture of a patient's seizure activity over extended periods. Although scalp EEG systems have long been the cornerstone of seizure monitoring, their use is limited to short‐term applications, and even with repeat or high‐acuity health care utilization, they cannot offer true long‐term continuous seizure detection. Recent advances have focused on the development of implantable continuous EEG monitoring (iCEM) systems, which aim to provide reliable, long‐term data for patients with epilepsy outside of the clinical setting.^14^, ^15^

The Minder iCEM system is one such device that offers the potential for continuous, real‐time monitoring of brain activity.^16^ Signal quality and long‐term signal stability are fundamental prerequisites for the clinical utility of any implantable EEG monitoring system, as the device must maintain consistent, interpretable recordings across extended ambulatory monitoring periods. The current investigation aims to compare the signal quality and noise characteristics of the Minder iCEM system to those of the standard scalp EEG system during co‐monitored periods. Analyses comparing Minder EEG findings to seizure diaries, patient management decisions, and clinical outcomes over the 6‐month implantation period are reported in our primary sUb‐scalp Monitoring ePileptic seIzuREs (UMPIRE) study.^16^

## 2 MATERIALS AND METHODS

This study was approved by St. Vincent's Ethics and Governance (Project ID 57264) and registered on the Australian New Zealand Clinical Trials Registry (ACTRN12619001587190).

Detailed information about the study design is available in Halliday et al.^16^ The cohort comprised 26 adults (mean age 45 years, range 23–71; 50% assigned female sex at birth) with focal epilepsy (88.5%; *n* = 23/26) or idiopathic generalized epilepsy (11.5%, *n* = 3/26). Seizure‐focus locations included temporal, frontal, parietal, posterior quadrant, and multifocal regions, and included pathology, that is, periventricular nodular heterotopia, polymicrogyria, posttraumatic epilepsy, and hypothalamic hamartoma, as well as nonlesional cases. Mean baseline seizure frequency was 10 seizures per month (range 1–60). Patients were taking a mean of 3 current anti‐seizure medications (range 1–6), with a mean of 3 previously failed medications (range 0–7). Full individual‐level characteristics including etiology and seizure semiology are provided in Halliday et al.^16^ and in this article in Tables S1 and S2.

**FIGURE 1:**
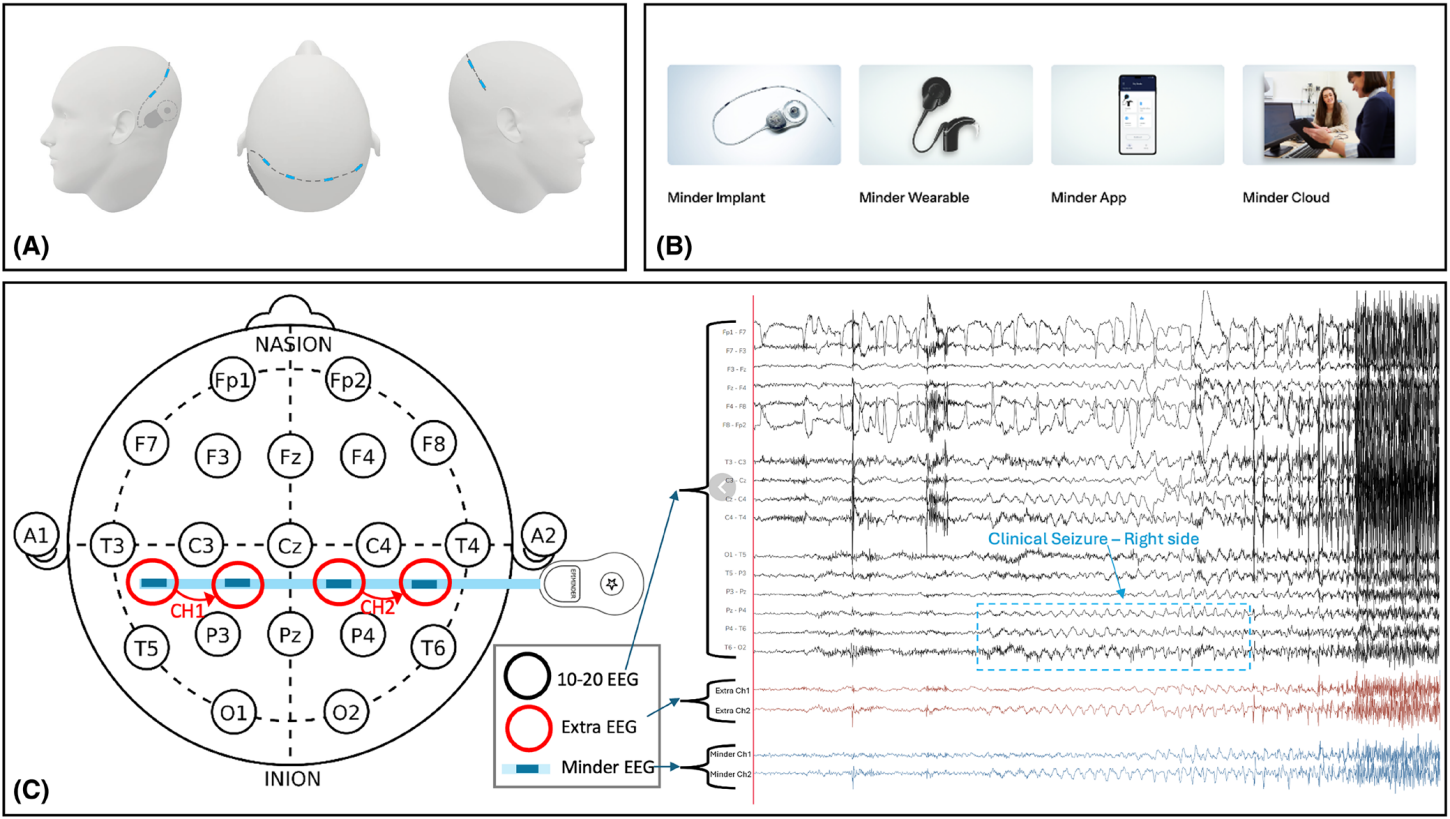
(A) Diagram of the Minder subscalp electrode placement. (B) An illustration of the Minder sub‐scalp EEG monitoring system including the surgical tools, Minder Implant, Minder Wearable, Minder App, and Minder Cloud. (C) Diagram of the 10–20 electrode placement along with the four Minder electrodes and four “extra” scalp electrodes placed over Minder sub‐scalp electrodes. The Minder and extra electrodes each generate two differential EEG channels (Channel 1 and Channel 2). EEG recordings are shown corresponding to the electrode placements.

The study involved subjects who were implanted with the Minder Implant across four clinical sites. The Minder iCEM system consists of the Minder Implant, Minder Wearable, Minder App, and Minder Cloud. The Minder Implant consists of a transmitter unit attached to a 253 mm electrode lead with four recording contacts placed just posterior to the vertex in an ear‐to‐ear position in the subgaleal space. The external Minder Wearable receiver streams EEG signals to the Minder App on an Android phone via Bluetooth. The Minder App periodically transmits EEG signals to the Minder Cloud, a cloud‐based platform that enables remote EEG export and reviewing using third‐party software. The implant location and the Minder system are shown in Figure 1A,B.

At both 4 and 24 weeks post‐implantation, subjects participated in 7‐day video and EEG monitoring (VEM) session using the international 10–20 scalp EEG montage. Scalp EEG signals were recorded using a Compumedics Siesta amplifier (Melbourne, Australia) in combination with the ProFusion EEG software. Four extra scalp electrodes were placed directly over the Minder sub‐scalp electrode locations to allow direct comparison between the surface and sub‐scalp EEG signals. An example of this co‐monitoring can be seen in Figure 1C (10–20 EEG, Minder EEG, and extra EEG). All scalp and Minder EEG data were uploaded to the Seer Cloud online EEG viewing/annotation platform, with Minder EEG data time‐aligned to the corresponding scalp EEG. Scalp video‐EEG co‐monitoring was performed at 4 weeks post‐implantation to assess signal quality following device stabilization, and at 24 weeks to evaluate the long‐term functional integrity of the Minder system. Comparison of signal quality between these two timepoints was a pre‐specified secondary objective of the study.

### 2.1 Comparison of co‐monitored EEG signals

In addition to comparing co‐monitored seizures, we also compared 10‐s EEG segments of spontaneous brain activity and artifacts such as chewing, sleep spindles, and interictal epileptiform events/discharges (IEEs). These signals represent a range of different physiological signal types with different frequencies.

These events were first identified by a U.S. board‐certified epileptologist (T.M.G.) based on reviewing 10–20 EEG data. T.M.G. continuously reviewed and manually labeled a total of 1460 h (60 days) of simultaneously acquired subscalp and scalp EEG to identify 541 candidate signals based on the 10–20 scalp EEG recording alone, from which 116 were randomly selected for blinded reviewer rating by two additional board‐certified epileptologists. A database of 541 signals was created that contained both limited montage scalp and Minder EEG recording of each segment. The total 116 randomly selected 10‐s EEG segments containing samples of chewing artifacts (42), sleep spindles (38), or IEEs (36) were chosen from this database for human review; 66 segments of EEG were from 4 weeks after implantation, and 50 segments of EEG at 24 weeks after implantation. Each segment consisted of both an extra limited montage scalp and Minder EEG clip; all segments were rated by three reviewers (696 ratings total). U.S. board‐certified epileptologists (J.S., P.K., and R.K.) independently scored each segment from each event type (chewing, sleep spindles, and IEEs) on a scale of 1 to 5, with 1 being no appreciable signal and 5 being a clear signal, and they were blinded to whether the signals were from the scalp or Minder electrodes. To compare signal clarity between scalp and Minder, we conducted a two‐factor analysis of variance (ANOVA) with replication. The ANOVA included system (scalp vs Minder) and reviewer as fixed factors, with multiple clips per system × reviewer cell providing replication.

### 2.2 Seizure co‐monitoring identification and rating

Co‐monitored seizures were identified using the 10–20 EEG review, facilitated by cloud‐based software (Persyst, Solana Beach, CA, USA) with the P14 seizure‐detection algorithm, or by patient‐reported events. Seizures flagged by the Persyst P14 algorithm were labeled and imported into Seer for human review. All candidate seizures—whether algorithm identified or patient reported—were subsequently reviewed independently by two board‐certified epileptologists. A third reviewer was consulted when the primary reviewers disagreed. Only seizures confirmed by consensus of at least two reviewers were included in the analysis for rating. Minder EEG, 10–20 EEG, and extra EEG were reviewed using the Seer viewer. Seizures were rated from 1 to 5, with 1 being no appreciable seizure and 5 being a clear seizure, by two board‐certified epileptologists (C.E., E.C., R.R., and T.M.G.). Ratings were compared for statistical significance using the Wilcoxon signed‐rank test.

### 2.3 Statistical comparison of EEG signals

The signal quality between the Minder and scalp recording was also objectively compared using statistical signal‐processing methods.

Spectral coherence (mscohere function in Matlab by Mathworks, Portola Valley, CA, USA) was used to determine whether the Minder and Scalp signals were similar (“coherent”) across a range of different frequencies. Correlation analysis using Pearson's correlation coefficient (corrcoef function in Matlab) was used to determine overall signal similarity, generating a single statistic for the similarity of each pair of signals over time. Both coherence and correlation were normalized from 0 (no relationship between the signals) to 1 (perfect linear relationship). Signals were unfiltered for coherence but were pre‐filtered from .5 to 70 Hz prior to correlation to reflect typical EEG viewer default settings.

Both spectral coherence and correlation were calculated on each pair of raw signals from both channels (MinderChannel1 vs ScalpChannel1, MinderChannel2 vs ScalpChannel2), yielding 216 analysis pairs (232, or 2 × 116, pairs were not obtained because raw signals were unavailable for 8 segments/16 analysis pairs for technical reasons).

## 3 RESULTS

### 3.1 Comparison of co‐monitored EEG signals

**FIGURE 2:**
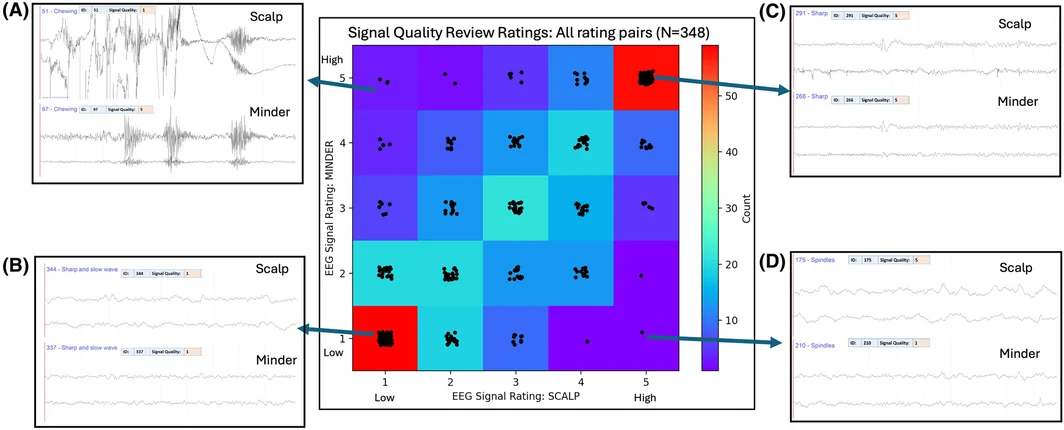
Heat maps comparing the Minder signal quality ratings to the extra scalp signals. Reviewer signal quality rating scale used was: 1 = Poor EEG signal, not readily recognized; 2 = EEG signal recognized, evaluation difficult; 3 = EEG signal recognized, evaluation possible but not optimal; 4 = EEG signal clear, evaluation possible and close to optimal; and 5 = EEG signal clear, evaluation straightforward. (A–D) Example ratings for four, 10‐s of EEG signals of Minder and extra scalp electrodes. (A) Signal rated 5 for Minder electrodes and 1 for scalp electrodes. (B) Signal rated 1 for both Minder and scalp electrodes. (C) Signal rated 5 for both Minder and scalp electrodes. (D) Signal rated 1 for Minder electrodes and 5 for scalp electrodes.

Three independent reviewers rated 116 total randomly selected segments for review, yielding 348 scalp vs Minder rating pairs (198 at 4 weeks, 150 at 24 weeks). Heat maps comparing the three independent human reviewer ratings of the Minder to the four extra scalp electrodes at 4 and 24 weeks are shown in Figure 2. Figures A–D show representative examples of the signal ratings in the heat map. The majority of ratings fall close to the diagonal line that indicates Minder‐scalp rating equivalence (83% of Signal Quality ratings between Minder and scalp electrodes differed by ≤1; 97% differed by ≤2). The Wilcoxon signed‐rank test showed no statistically significant difference between Minder vs scalp signal quality ratings overall (*p* = .973), at 4 weeks (*p* = .754) or at 24 weeks (*p* = .727). A two‐factor ANOVA with replication revealed no significant effect of system on EEG clarity (*F*(1,690) = .017, *p* = .90). There was a significant effect of rater (*p* < .001), reflecting the expected variability among individual raters.

There was no statistically significant difference between the Minder signal clarity ratings at 4 vs 24 weeks using a Wilcoxon rank‐sum test (*p* = .95) for subjects who had completed 24 weeks at the time the data analyzed (5 subjects, *N* = 267 rating).

**FIGURE 3:**
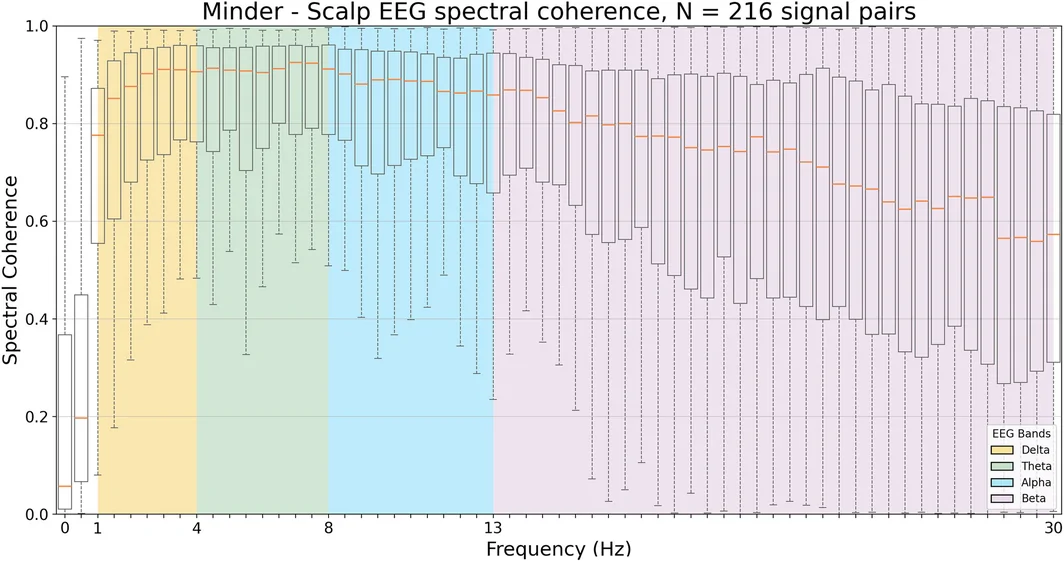
Spectral coherence of 216, 10‐s EEG clips rated by the reviewers. Horizontal axis is frequency (Hz) in steps of .5 Hz. Frequency bands are color coded to indicate delta, theta, alpha, and beta EEG frequency bands. Vertical axis is spectral coherence shown as boxplots between Minder and scalp EEG ranging between 0 and 1. Orange line shows the median, box edges are 25th and 75th percentiles, whiskers extend to most extreme data points not considered outliers, and blue dots are outliers.

**FIGURE 4:**
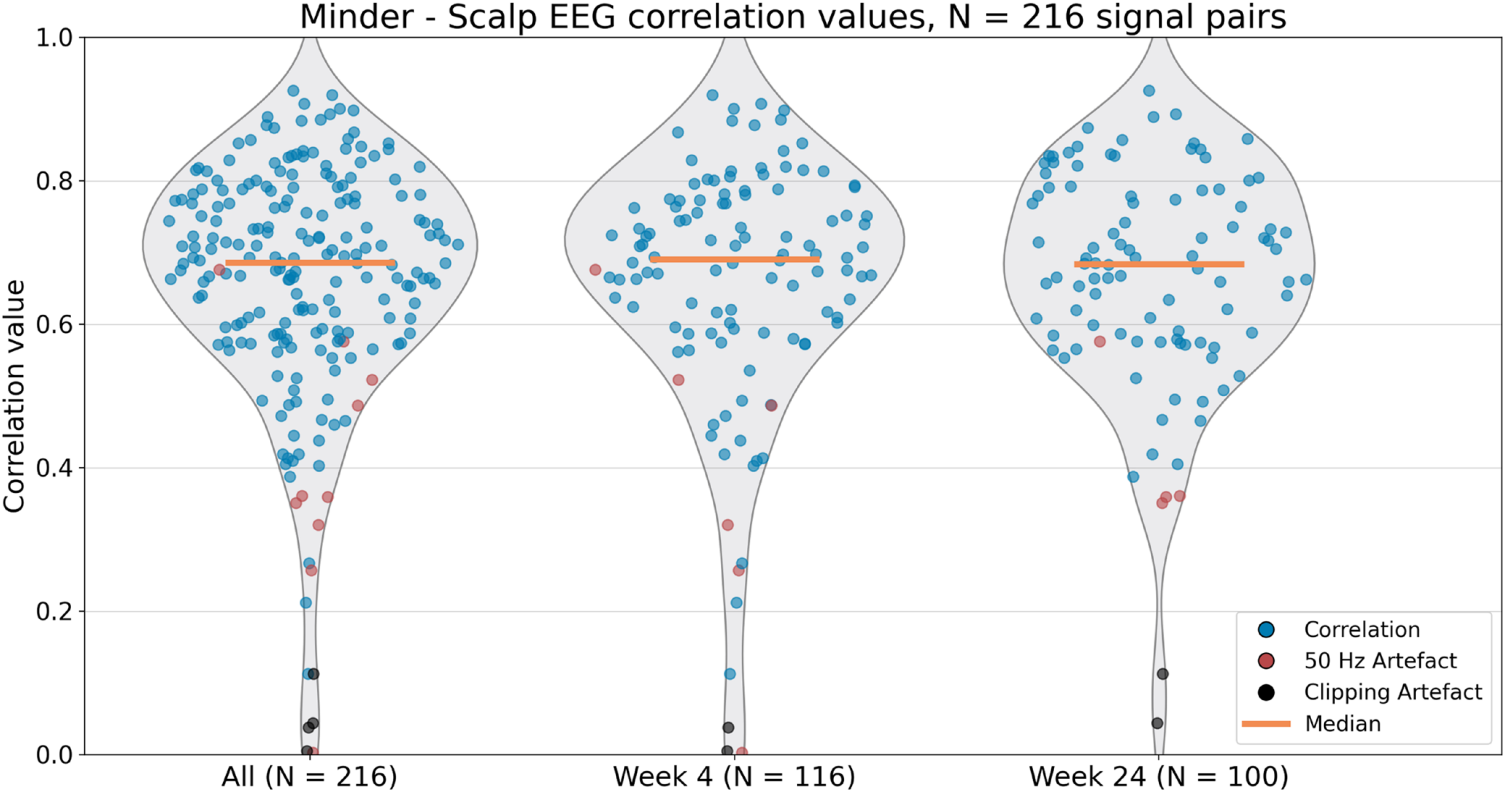
Violin with strip plot of correlation between Minder and scalp EEG (both sets of EEG recordings were filtered with a .5 Hz high‐pass filter, 50 Hz notch filter, and 70 Hz low‐pass filter of as per typical EEG review software). The three plots represent all 10‐s EEG segments rated (*n* = 216), those only at Week 4 (*n* = 116) and those only at Week 24 (*n* = 100). The *y*‐axis is normalized Pearson's correlation between Minder and scalp EEG. Datapoints show correlation values. Correlation values shown by the red circle indicate when one (or more) of the EEG signal pairs exhibited significant 50 Hz mains interference artifacts (*N* = 12). Those shown by the black circle indicate high‐amplitude artifacts (*N* = 4). Two values had both high‐amplitude and 50 Hz artifacts. Median values for each violin plot are indicated.

The results of the spectral coherence and Pearson's correlation coefficient of the 10‐s EEG segment ratings are found in Figures 3 and 4.

A high coherence between Minder and scalp signals across most of the clinically relevant delta‐beta frequency range (median coherence >.55 from 2–30 Hz, > .7 from 2–23 Hz) is shown in Figure 3. The 1–2 Hz median coherence is lower (.15), which is expected due to the Minder implant's low‐frequency cutoff being higher than typical scalp EEG (see Discussion). Lower coherence in the high beta range may be related to reduced muscle artifact in the Minder.

A high correlation between Minder and scalp EEG (median correlation overall = .686, Week 4 = .691, Week 24 = .684) is shown in Figure 4. Figure 4 also shows correlation points where one or more of the signals was impacted by 50 Hz main power artifacts (defined as 50 Hz power >20% of total spectral power before filtering; red circles) or high‐amplitude artifacts (defined as amplitude exceeding 500 μV for >5% of the 10s window; black circles). Fourteen of 216 points had at least one form of severe artifact. Of the points with very low correlation values (<.2), 83% (5 of 6) exhibited either artifact.

In addition, there was no statistically significant difference between correlation values at 4 vs 24 weeks (*p* = .986). These results are consistent with the reviewer ratings seen in Figure 2.

### 3.2 Seizure co‐monitoring identification and rating

In addition to EEG segments, 26 seizures were also identified during the co‐monitoring period from eight subjects (12 found by diary, 14 found by the Persyst 14 algorithm without a corresponding patient diary entry). Of the 26 co‐monitored seizures from eight subjects, seizures arose in patients with both focal and generalized epilepsy. Seizure types included focal aware seizures, focal impaired awareness seizures, and generalized seizures. Full per‐patient ictal semiology and clinical characterization are provided in Halliday et al.^16^

Possible reasons for non‐reporting include loss of awareness, purely electrographic events with no perceptible clinical manifestation, nocturnal seizures occurring during sleep, and postictal cognitive impairment precluding timely self‐report. This rate of underreporting is consistent with prior studies demonstrating that a substantial proportion of seizures are not recognized by patients and not captured in self‐reported diaries.

**FIGURE 5:**
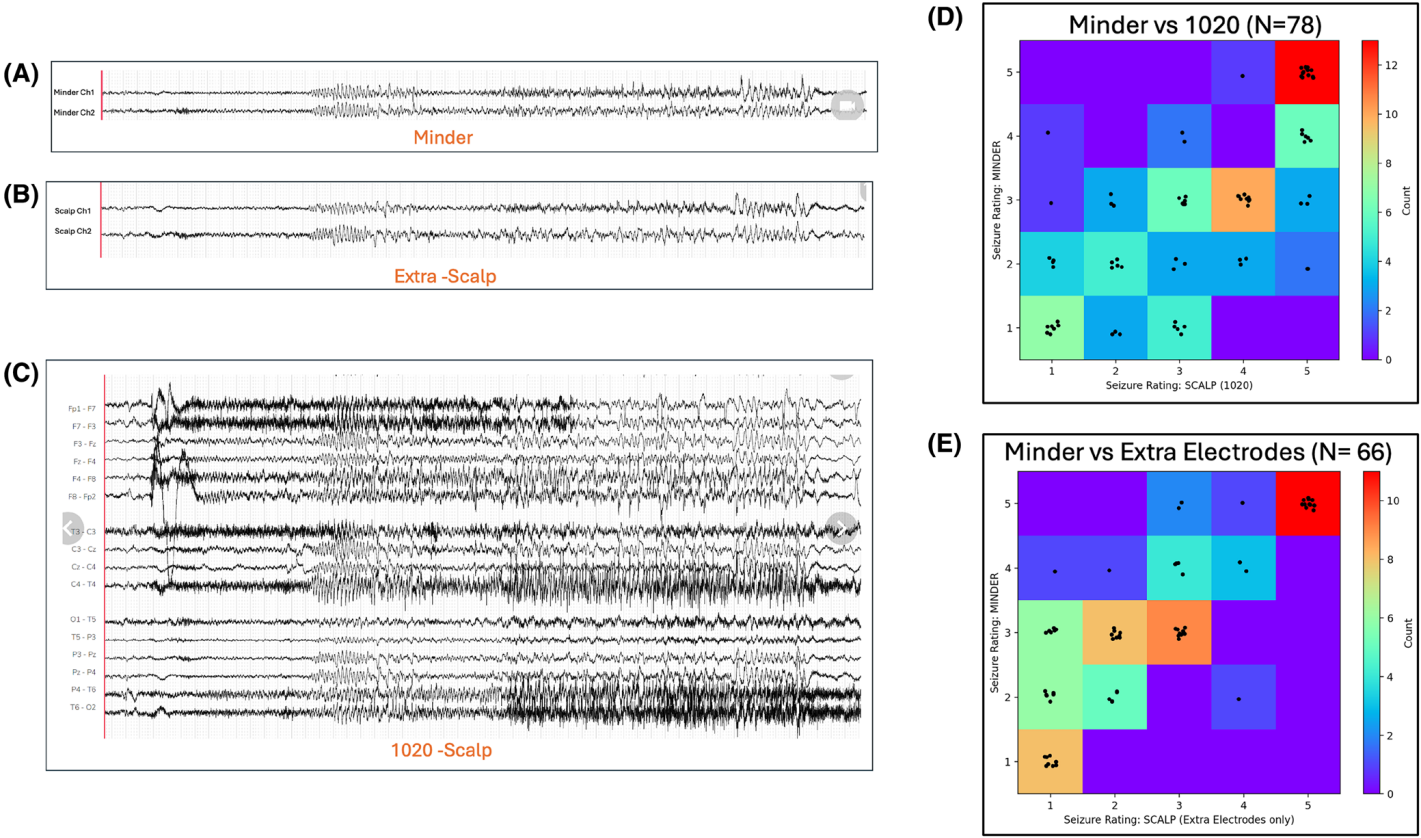
Examples of the seizure recording rated by the reviewers, (A) the two Minder electrode channels, (B) the two “Extra” scalp electrode channels, and (C) the full 1020 set of scalp electrodes. Heat maps comparing all 26 seizure ratings using the (D) Minder electrodes to those using 1020 electrodes and the seizure ratings using the (E) Minder electrodes to those using four extra scalp electrodes. Seizure rating scale: 1–5, where 1 = Very unclear, 5 = Clear, Confident seizure.

All 26 seizures found on the 10–20 scalp electrodes were also seen on the Minder electrodes. Four seizures identified on the full 10–20 scalp EEG were not clearly appreciated on the two‐channel extra scalp electrode montage, chiefly due to artifact contamination. Reduced sensitivity of this limited montage to deeper or more lateral seizure sources may also have contributed. Thus only 22 seizures were compared between the extra scalp electrodes and the Minder. Figure 5A–C shows the same seizure recorded by (A) the Minder electrodes, (B) the extra four electrodes, and (C) the 1020 electrodes. Heat maps were used to compare the three independent human reviewer ratings from all visible seizures. Figure 5D shows a heat map comparing the seizure ratings between the Minder and 10–20 electrodes, and Figure 5E shows a heat map comparing the seizure ratings between the Minder and extra electrodes.

A statistically significant difference was found between the rating of the Minder seizures compared to the 10–20 seizures (mean 10–20 rating = 3.3 + 1.5, Minder rating = 2.9 + 1.4, *p* < .001) and between the ratings of the Minder seizures compared to the extra electrodes (mean extra scalp rating = 2.6 + 1.4, Minder rating = 3.1 + 1.4, *p* < .001), meaning that the seizures were seen most clearly on 10–20, followed by Minder, and then by the extra scalp electrode.

**FIGURE 6:**
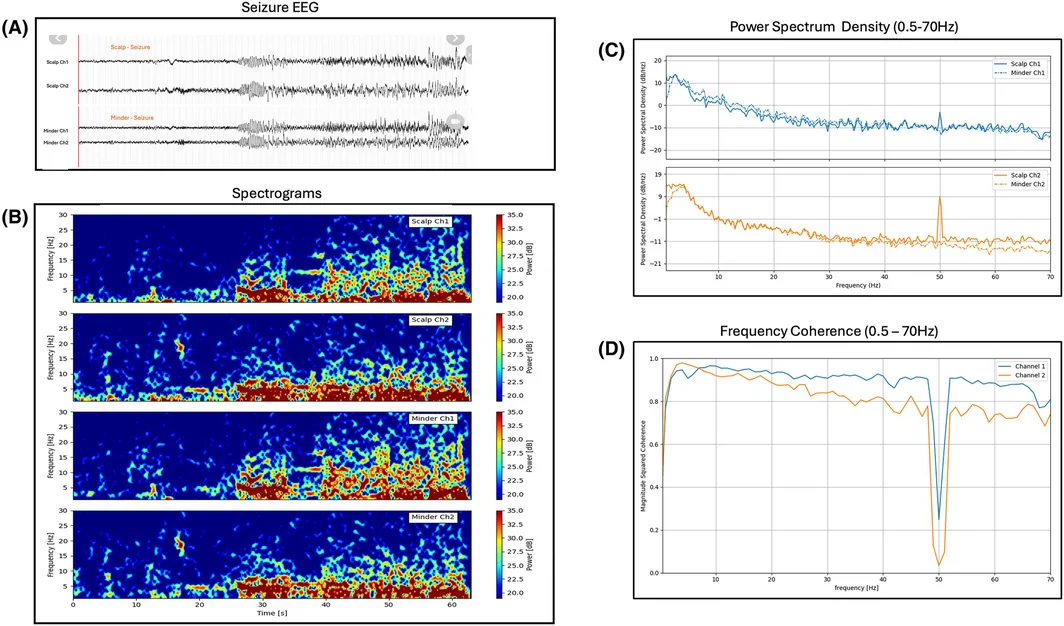
Results of statistical analysis of a seizure recording from one subject. (A) Graphs showing a 60‐s segment of the seizure, (B) the spectrograms of Channel 1s and 2 from both the scalp and Minder recordings, (C) the power spectral density of Channels 1 (blue) and 2 (orange) of Minder (dotted) and surface (solid) EEG recordings. (D) Graph of the frequency coherence plot comparing Minder EEG to the surface EEG.

Spectrograms from a seizure recorded from scalp and Minder electrodes are shown in Figure 6. Figure 6A shows the raw seizure segments from both Channel 1 and Channel 2 of the Minder and scalp electrodes. Figure 6B shows the spectrogram of each of the four channels. Figure 6C compares the power spectral density of the scalp and Minder channels, showing the strong congruence of frequency content over the frequency spectrum. Figure 6D compares the frequency coherence of the scalp and Minder channels, showing high similarity of frequency content and timing through the seizure, with notable exceptions for 50 Hz signal artifact.

## 4 DISCUSSION

The findings from this study provide insights into the viability of the Minder iCEM system for epilepsy management. The results were assessed both through expert reviewer ratings and quantitative EEG analysis, comparing the Minder EEG to the current gold standard of scalp EEG using the International 10–20 system.

### 4.1 Comparison of co‐monitored EEG signals

Our primary objective was to compare the signal quality of the Minder system based on expert human reviewers, as the ultimate use case for the system is with clinicians treating patients with suspected epilepsy. The interictal EEG signals were specifically chosen to demonstrate the frequency range and fidelity of the Minder Implant including interictal epileptiform events, spindles, and muscle artifact. The signal quality and noise level scores as rated by expert reviewers showed that these Minder interictal recordings were comparable to 10–20 scalp EEG. When there was a significant difference between Minder and scalp ratings, noise introduced from the scalp electrodes was noted.

We aimed to demonstrate that the long‐term functional integrity of the Minder system showed that signal quality was maintained at 24 weeks. Our Pearson's correlation demonstrated similar ratings between the two EEG systems at both Week 4 and Week 24. Subgroup analysis demonstrated that some of the differences in ratings were attributable due to 50 Hz artifact and motion artifact in scalp electrodes and not due to Minder performance. The minimal degradation in signal quality reinforces the potential of the Minder system for long‐term use.

Our secondary goal was using quantitative EEG tools such as spectral coherence analysis to objectively compare signals captured on the Minder system compared to 10–20 scalp EEG. The Minder system demonstrated high coherence at most clinically relevant frequencies (delta‐gamma frequencies). This was also demonstrated with the power spectral analysis that found most of the power is between 0 and 30 Hz (delta, theta, alpha, and beta bands). The consistency in scores between modalities indicates that Minder may offer a reliable alternative for prolonged monitoring without significantly compromising quality. However, subtle differences were observed, potentially attributable to factors such as electrode geometry and amplifier characteristics. The sub‐scalp recordings demonstrated reduced sensitivity at lower frequencies (≤1 Hz), likely due to the Minder device's higher low‐frequency cutoff (~1.5 Hz) compared to the Compumedics Siesta (.15 Hz). This reduced sensitivity may impact detection at very low frequencies, but in our study it was not demonstrated to impede human review of representative EEG signals. Some low coherence values present at higher frequencies may be related to the rarity of high‐ frequency components being present in some of the signals (e.g., spindles). When frequency coherence values are calculated on a chewing signal (containing high‐frequency components) the coherence is very high.

### 4.2 Seizure co‐monitoring identification and rating

We also demonstrated that human reviewers were able to detect all seizures captured on 10–20 EEG on the Minder system. The 10–20 scalp EEG did receive statistically higher seizure clarity ratings compared to the Minder system, which is to be expected given the 10–20 scalp EEG is the gold standard with greater spatial resolution and the added benefit of video to confirm electrographic findings. Minder, however, demonstrated statistically higher seizure clarity readings when compared to limited montage scalp EEG at the vertex, which was likely attributable to the benefit of reduced motion artifact, less 50 Hz interference, and the absence of electrode degradation seen with extended scalp EEG use.

Objective correlation analysis between sub‐scalp and scalp EEG recordings with power spectrum density and frequency coherence further supported the close relationship between signal quality and noise levels.

The Pearson's correlation of signals between .5 and 70 Hz indicated a proportional relationship with expert ratings, suggesting that sub‐scalp EEG can reliably capture meaningful brain activity when signal quality is high.

### 4.3 Limitations

It is important to acknowledge certain caveats in this study. The Minder electrode lead is positioned posterior to the vertex and does not provide direct coverage of the temporal pole or inferior frontal cortex — regions that represent the most common locations of focal seizure onset. Despite this, in our patient cohort, all seizures captured on 10–20 EEG were also visualized on Minder. Although seizures from these regions may propagate sufficiently to be detectable at the vertex, this cannot be assumed for all seizure types, and detection sensitivity may be reduced for seizures with restricted propagation such as focal aware seizures with onset distant from the vertex. This spatial limitation should be considered when applying subscalp EEG to patients with these epilepsy subtypes, and should inform the design of future electrode configurations. In addition, although all seizures captured on 10–20 were visualized on Minder, this does not directly translate to seizure sensitivity as the study was designed to evaluate for signal and seizure clarity on the minder device.

The reduced electrode array in sub‐scalp EEG represents a trade‐off between spatial resolution and continuous monitoring. Although scalp EEG offers greater spatial resolution, sub‐scalp EEG prioritizes temporal continuity. Finally, although comparison of limited montage scalp EEG and Minder was blinded, it was not possible to perform a blinded comparison of 10–20 and Minder.

## 5 CONCLUSION

The findings of this feasibility study evaluating the signal quality of the Minder iCEM system support its ability as a promising tool for long‐term bilateral subscalp EEG monitoring. Interictal signal quality was statistically equivalent to limited‐montage scalp EEG. Although this was not a study of seizure‐detection sensitivity, all seizures detected on full 10–20 scalp EEG were also identifiable on the Minder system. The 10–20 system did receive statistically higher seizure clarity ratings than the Minder, which is expected given its greater electrode coverage. Although the full 10–20 system retains advantages in spatial resolution, this study demonstrates that the Minder system achieves consistent signal quality over extended monitoring periods and offers value as a stable, continuous long‐term monitoring solution in settings where short‐term ambulatory scalp EEG is impractical or insufficient. The clinical impact of long‐term Minder monitoring on epilepsy management will need to be evaluated in larger prospective studies.

A key advantage of sub‐scalp EEG lies in its stability over prolonged periods. Minimal signal degradation at 4‐week and 24‐week intervals underscores its capacity for reliable long‐term use, which is particularly relevant in epilepsy management where intermittent short‐term recordings may fail to capture the full burden of seizure activity. In contrast, scalp EEG requires repeated application and is prone to signal loss due to electrode displacement or skin impedance changes over time.

The Minder system also demonstrated reduced susceptibility to certain artifacts compared to limited‐montage scalp EEG. Scalp recordings in this study were more frequently affected by powerline interference and motion artifact, both of which contributed to lower correlation values in a subset of segments. Sub‐scalp electrodes, shielded by subscalp tissue, appear to offer some protection against these sources of noise.

Although the reduced electrode array in sub‐scalp EEG limits cortical coverage compared to the full 10–20 system, this reflects an intentional trade‐off in favor of continuous long‐term data collection. For many clinical questions in epilepsy—including accurate seizure counting, medication response assessment, and differentiation of epileptic from non‐epileptic events—longitudinal coverage may be of greater value than spatial sampling in the right clinical scenario.

In conclusion, the Minder system represents an advancement in epilepsy monitoring, combining long‐term stability, reduced susceptibility to artifacts, and enhanced sampling continuity. These findings position subscalp EEG as a feasible and promising complement to scalp EEG for continuous long‐term monitoring, particularly in ambulatory settings. Broader clinical adoption will require prospective studies in larger and more diverse cohorts to characterize performance across epilepsy subtypes and to evaluate impact on patient management.

## AUTHOR CONTRIBUTIONS

*Investigation*: Taneeta Mindy Ganguly, Ramya Raghupathi, Colin Ellis, Erin C. Conrad, Amy J. Halliday, Lisa Gillinder, Alan Lai, Udaya Seneviratne, Patrick Kwan, Piero Perucca, Aileen McGonigal, and Mark J. Cook. *Study design*: Mark J. Cook, Holly Fontenot, and Tracy Cameron. *Writing—original draft*: Taneeta Mindy Ganguly and Andrew Niemiec. *Writing—review and editing*: Tracy Cameron, Alan Lai, Erin C. Conrad, Colin Ellis, and Mark J. Cook. *Data curation*: Tracy Cameron, Alan Lai, Sara Vogrin, Holly Fontenot, and Andrew Niemiec.

## CONFLICT OF INTEREST STATEMENT

T.C. is VP of Clinical & Regulatory, A.N. is Principal Product Engineer, H.F. is Director of Clinical Trials, and M.J.C. is CMO of the Epiminder LTD. K.M., A.N., R.R., T.M.G., C.E., and E.C.C. were employed by the Epiminder LTD. M.J.C., A.N., and T.C. hold stock options in Epiminder LTD. The remaining authors have no conflicts of interest. We confirm that we have read the Journal's position on issues involved in ethical publication and affirm that this report is consistent with those guidelines.

## Supporting information


Table S1.

## Data Availability

The data that support the findings of this study are available from the corresponding author upon reasonable request.
