# Supplementary material for: Feasibility and signal quality of the Minder® implantable continuous EEG monitoring® system compared to 10–20 scalp electrodes over extended monitoring periods
Source: Epilepsia. 2026 May 20;67(8):4234–44. doi: 10.1002/epi.70290 (PMC13525562; doi:10.1002/epi.70290)
Supplement: Supplementary file 1 [file EPI-67-4234-s001.docx]

**Supplemental Table 1. Cohort summary.**

| **Characteristic** | **Total (n=26)** |
| --- | --- |
| **Female, n (%)** | **13 (50%)** |
| **Age at enrolment, mean (range)** | **45 (23-71)** |
| **Epilepsy type, n (%)** |  |
| **Focal** | **23 (88.5%)** |
| **Generalised** | **3 (11.5%)** |
| **Number of ASMs, mean (range), (n=25)** | **3 (1-6)** |
| **Number of previously failed ASMs, (n=21)** | **3 (0-7)** |
| **Baseline seizure frequency, (n=23)** | **10 (1-60)** |

**Abbreviations: ASM antiseizure medication.**

**Supplemental Table 2. Summary of implanted patients.**

| **Subject ID** | **Gender** | **Age** | **Seizures/month** | **Pathology** | **Basis for localisation** | **VEM** | | | | | | **Seizure diary** | | | | | |
| --- | --- | --- | --- | --- | --- | --- | --- | --- | --- | --- | --- | --- | --- | --- | --- | --- | --- |
|  |  |  |  |  |  | **Reported events** | **Seizures: reported** | **Seizures: unreported** | **Seizures: total** | **Sub-scalp/scalp (%)** | **Reported/total (%)** | **Reported events** | **Reported + EEG** | **EEG/reported (%)** | **EEG + seizure** | **Seizures/reported (%)** |  |
| **106** | **M** | **45** | **8** | **IGE** | ***na*** | **1** | **0** | **1** | **1** | **100** | **0** | **14** | **11** | **79** | **3** | **27** |  |
| **202** | **F** | **30** | **25** | **IGE** | ***na*** | **17** | **2** | **1** | **3** | **100** | **100** | **59** | **26** | **44** | **3** | **12** |  |
| **301** | **F** | **46** | **9** | **IGE** | ***na*** | **3** | **0** | **0** | **0** | ***na*** | ***na*** | **25** | **12** | **48** | **1** | **8** |  |
| **107** | **M** | **25** | **60** | **Left posterior quadrant epilepsy** | **EEG** | **no VEMs** | | | | | | **0** | **na** | **na** | **na** | **na** |  |
| **108** | **M** | **71** | **1** | **Left TLE, lesion negative** | **EEG + semiology** | **4** | **3** | **1** | **4** | **100** | **67** | **5** | **2** | **40** | **1** | **50** |  |
| **112** | **M** | **51** | **2** | **Left temporal heterotopic grey matter** | **EEG + MRI** | **1** | **0** | **0** | **0** | ***na*** | ***na*** | **19** | **14** | **74** | **6** | **43** |  |
| **116** | **M** | **23** | **20** | **Left perisylvian epilepsy** | **EEG + semiology** | **10** | **0** | **0** | **0** | ***na*** | ***na*** | **13** | **10** | **77** | **7** | **70** |  |
| **118** | **F** | **50** | **2** | **Left anterior quadrant epilepsy** | **EEG** | **0** | **0** | **0** | **0** | ***na*** | ***na*** | **14** | **5** | **36** | **0** | **0** |  |
| **102** | **M** | **60** | **12** | **Right parietal polymicrogyria** | **EEG + MRI** | **6** | **0** | **0** | **0** | ***na*** | ***na*** | **39** | **29** | **74** | **11** | **38** |  |
| **105** | **F** | **47** | **3** | **Right TLE, lesion negative** | **EEG + semiology** | **0** | **0** | **0** | **0** | ***na*** | ***na*** | **6** | **4** | **67** | **0** | **0** |  |
| **109** | **M** | **48** | **2** | **Right frontal encephalomalacia** | **EEG + MRI** | **0** | **0** | **1** | **1** | **100** | **0** | **2** | **0** | **0** | **0** | **0** |  |
| **111** | **F** | **38** | **nd** | **Right TLE** | **Prior resection** | **2** | **0** | **0** | **0** | ***na*** | ***na*** | **53** | **42** | **79** | **16** | **38** |  |
| **115** | **M** | **65** | **2** | **Right TLE, lesion negative** | **EEG + semiology** | **0** | **0** | **0** | **0** | ***na*** | ***na*** | **0** | **na** | **na** | **na** | **na** |  |
| **201** | **F** | **25** | **4** | **Right temporal pole epilepsy** | **Prior resection** | **14** | **0** | **0** | **0** | ***na*** | ***na*** | **24** | **17** | **71** | **1** | **6** |  |
| **205** | **F** | **42** | **3** | **Right TLE, lesion negative** | **EEG + semiology** | **4** | **0** | **0** | **0** | ***na*** | ***na*** | **17** | **12** | **71** | **0** | **0** |  |
| **101** | **F** | **49** | **20** | **Bilateral PVNH** | **MRI** | **10** | **5** | **4** | **10** | **100** | **36** | **73** | **44** | **60** | **19** | **43** |  |
| **114** | **M** | **59** | **5** | **Multifocal post-traumatic epilepsy** | **EEG + MRI** | **3** | **0** | **1** | **1** | **100** | **0** | **13** | **9** | **69** | **3** | **33** |  |
| **204** | **M** | **38** | **5** | **Temporal lobe epilepsy, not lateralised** | **MRI (bilateral HS)** | **1** | **0** | **0** | **0** | ***na*** | ***na*** | **21** | **11** | **52** | **0** | **0** |  |
| **206** | **F** | **54** | **8** | **Bilateral perisylvian polymicrogyria** | **MRI** | **1** | **0** | **0** | **0** | ***na*** | ***na*** | **9** | **3** | **33** | **3** | **100** |  |
| **404** | **F** | **39** | **5** | **Temporal lobe epilepsy, not lateralised** | **MRI (bilateral HS)** | **6** | **1** | **3** | **4** | **100** | **20** | **43** | **31** | **72** | **15** | **48** |  |
| **405** | **F** | **54** | **nd** | **Bifrontal encephalomalacia** | **MRI** | **no VEMs** | | | | | | **No diary** | | | | |  |
| **207** | **M** | **51** | **6** | **Temporal lobe epilepsy, not lateralised** | **Semiology** | **1** | **0** | **0** | **0** | ***na*** | ***na*** | **48** | **27** | **56** | **15** | **56** |  |
| **401** | **M** | **29** | **2** | **Temporal lobe epilepsy, not lateralised** | **SEEG + semiology** | **1** | **0** | **2** | **2** | **100** | **50** | **23** | **7** | **30** | **4** | **57** |  |
| **403** | **M** | **55** | **nd** | **Temporal lobe epilepsy, not lateralised** | **MRI (bilateral HS)** | **1** | **0** | **0** | **0** | ***na*** | ***na*** | **13** | **9** | **69** | **1** | **11** |  |
| **117** | **F** | **38** | **6** | **Temporal lobe epilepsy, not lateralised** | **Semiology** | **4** | **0** | **0** | **0** | **na** | **na** | **31** | **30** | **97** | **6** | **20** |  |
| **104** | **F** | **44** | **20** | **Hypothalamic hamartoma** | **MRI** | **5** | **0** | **0** | **0** | ***na*** | ***na*** | **36** | **22** | **61** | **0** | **0** |  |

**Patients with seizures captured during VEM comonitoring periods are shown in bold.**

**Abbreviations: ATL anterior temporal lobectomy. EEG electroencephalogram. IGE idiopathic generalised epilepsy. na not applicable. nd not documented. MRI, magnetic resonance imaging. PVNH periventricular nodular heterotopia. SEEG, stereo-electroencephalography. TLE temporal lobe epilepsy. VEM video-EEG monitoring. XRT radiotherapy.**
